# Dental Erosion in Competitive Swimmers and Preventive Treatments: An In Vitro Study

**DOI:** 10.3390/dj12090289

**Published:** 2024-09-11

**Authors:** Riccardo Favero, Marianna Nicetto, Martina Barone, Alessandra Dorigotti, Andrea Volpato, Vincenzo Tosco

**Affiliations:** 1 Unit of Maxillofacial Surgery, Department of Neurosciences, University of Padua, Via Giustiniani 2, 35121 Padua, Italy; rickyfavero@msn.com (R.F.); mariannanicetto96@gmail.com (M.N.); martinabarone4@gmail.com (M.B.); alessandra.dorigotti@gmail.com (A.D.); andrea.volpato1@gmail.com (A.V.); 2 Department of Clinical Sciences and Stomatology (DISCO), Università Politecnica delle Marche, 60126 Ancona, Italy

**Keywords:** dental erosion, swimmers, preventive dentistry, athletes oral health

## Abstract

The aim of this in vitro study was to evaluate the effectiveness of highly concentrated fluoride products and remineralizing products (F-APC) in preventing erosive dental lesions in competitive swimming patients. A total of 48 teeth were extracted, preserved in saline solution and divided into three groups. In G1 (control group), each tooth was half-immersed in chlorinated pool water; in G2 (fluoride-treated group), after being totally immersed in chlorinated water, each tooth had half of its surface treated with a highly concentrated fluoride product once a week; in G3 (remineralization product-treated group), each tooth was totally immersed in chlorinated water and half of its surface was treated with a remineralizing product after each immersion. The study was conducted over a 4-week period, immersing the teeth for 4 h per day. In G1, statistically significant differences were observed in submerged versus non-submerged tooth surfaces at week 3 (T3) (*p* = 0.019) and week 4 (T4) (*p* = 0.0007), with four and eight surfaces showing erosive tooth wear (ETW), respectively. In G2, a difference was observed between fluoride-treated and non-fluoride-treated surfaces at T4 (*p* = 0.039), with three surfaces with ETW among the non-treated ones. In G3, the difference was observed between portions treated with F-APC and those not treated at T4 (*p* = 0.019), with four surfaces with ETW among the untreated ones. Chlorinated pool water is a potential erosive agent for teeth if water pH values reach a critical value. Treating the teeth surface with highly concentrated fluoride products, once a week for 5 min, and F-APC are effective in protecting teeth against tooth erosion.

## 1. Introduction

Erosive tooth wear (ETW) is defined as the pathological, progressive, and irreversible dissolution of dental hard tissue due to acids of non-bacterial origin [1,2]. It has been reported that 30% of the European population in the 18–35 age group has at least one tooth with ETW [3]. ETW can occur on both permanent and deciduous teeth, affecting either enamel alone or enamel and dentin, and may be localized or generalized. The most affected tooth surfaces are the palatal surfaces of the upper front teeth and occlusal surfaces of the lower first molars. At an early stage, ETW is clinically difficult to notice in both visual and tactile examination because it causes no discoloration or softening of the tooth surface. Also, very modest or completely absent symptoms are reported by the patient. Morphological changes appear when the erosive damage is at a more severe stage. The tooth surface is opaque, slightly polished, and flattened; in posterior teeth, a concavity is found on the cusps, while in anterior teeth, depressions and concavities can be seen in the smooth surfaces, which are typically wider and more extensive. Usually, the cervical third along the gingival margin remains intact. In later stages, the pulp may be visible in transparency, most commonly in the incisors [4]. The patient can refer to hypersensitivity and pain, and aesthetic and functional complications may occur.

During a lifetime, teeth are subjected to chemical and physical insults, which contribute to the erosive wear of the dental hard tissue. When an acid is ingested, the salivary pH drops to a critical value of 5.5, resulting in enamel surface dissolution. The loss of calcium and phosphate ions from the surface results in a loss of hardness and, if acid impacts persist over time, it makes the affected surfaces more susceptible to physical impacts [5]. Acid in the oral cavity can have intrinsic or extrinsic origins [6]. Intrinsic factors include gastro-esophageal reflux disease, eating disorders such as anorexia and bulimia nervosa, and pregnancy [7,8,9]. Exogenous acids may come from dietary acid intake, acidic drinks, fruit juices, energy drinks, wine, drug intake, or exposure in work settings [10]. 

Competitive swimmers are one of the sports categories most exposed to ETW. This is due to prolonged exposure to chlorinated swimming pool water [2,11]. Water chlorination is a necessary and effective method of water disinfection to prevent the occurrence of infectious diseases. Nevertheless, when chlorine compounds dissolve in water, they change the pH level: the presence of chlorine, if not properly buffered and monitored, drastically decreases the pH values of the water, leading up to very low (i.e., acid) and decalcifying levels [1,2,12]. Generally, swimming pool water is chlorinated using chlorine gas, which reacts with water and forms hypochlorous acid (HOCl, the germicidal agent) and hydrochloric acid (HCl, an unwanted product) [1]. The WHO sets a pH value standard for swimming pool water treated with chlorine disinfectants, which must be maintained between 7.2 and 7.8, although this value is not always maintained. For erosive lesions, enamel dissolution depends on both the solution pH and the concentration of the enamel’s constituent minerals (phosphate, fluoride, and calcium). Thus, a wide range of critical pH values, starting at 6.5, is found [13]. 

Common problems in swimmers’ oral cavities are dental pigmentation, caused by water-dissolved disinfectants, and dental erosion due to low pH values (2.8–4.5). As swimmers are constantly exposed to the acid erosive action of chlorine compounds, their teeth are susceptible to enamel erosion caused by the dissolution of hydroxyapatite crystals and the release of calcium ions [2]. Therefore, the high consumption of drinks among athletes can contribute to the risk of ETW [14].

The aim of this in vitro study is to evaluate the effectiveness of highly concentrated fluoride products and remineralizing products (F-APC) in preventing erosive dental lesions in competitive swimming patients.

## 2. Materials and Methods

### 2.1. Sample Collection

For the study, 85 human permanent teeth were collected from subjects aged between 18 and 30 years. Teeth were surgically extracted for orthodontic reasons at the Section of Stomatology of DISCO Department, Polytechnic University of Marche, Ancona, Italy. According to the Local Ethic Committee guidelines and the 1964 Helsinki declaration, informed consent was obtained from the subjects that were aware that their hard-dental tissues, as discard of the surgical procedures, would be used for research purposes. They were composed of maxillary and mandibular central incisors, canines, premolars, and molars. Those with carious lesions, fractures, restorations, or any other pathological condition were excluded. The final number of 48 teeth was reached and they were then divided into three groups, each consisting of 16 teeth. The teeth were thoroughly cleaned of tartar residue and soft tissue using scalers, low-intensity ultrasound, and yellow gum mounted on a micromotor to avoid minimal damage to the tooth structure. They were then stored in physiological saline. Afterwards, an initial evaluation was made by drying the teeth with an air-water gun and classifying them according to the BEWE classification (Table 1) [15].

### 2.2. Immersion in Chlorinated Water 

After the starting T_0_ analysis was completed, the teeth were divided into three groups and were immersed in chlorinated water simulating professional swimmer training hours. Over a period of 31 days, the teeth were immersed 4 h per day for 5 days each week. Immersion time amounted to a total of 95 h. The chlorinated water used to immerse the teeth was collected from a pool in Vicenza (Italy) every 2 days to ensure that the chemical values were always appropriate. The pH was checked daily with a litmus paper at pH 6.0, except for 4 days when it was pH 5.0. After the samples’ preparation, three different groups including 16 teeth each were formed, according to the test technique:

Control group (G1): A temporary Splintline resin base was made for each tooth in order to place the teeth in the same position inside a container. The container was then marked to indicate the level of water required to half-cover the prepared teeth inside. This was carried out in order to have a comparison with the non-immersed parts of the teeth, which were not exposed to the erosive agent. Once submerged, they were left for 4 h and then removed, rinsed, and placed inside a container with physiological water and bicarbonate to simulate the oral environment. The pH value was 7.0, checked daily with litmus paper. 

Group treated with remineralizing product (G2): The teeth were soaked inside a container with chlorinated water for 4 h. After that time, they were rinsed and the remineralizing product was applied to one half of the enamel surface of each tooth. The product used was Curasept Bioenamel Shock Action Mousse Caries Abrasion and Erosion (Curasept S.p.A., Saronno, VA, Italy), containing 1.450 ppm F- and Amorphous Calcium Phosphate (ACP). The mousse was applied according to the Curasept protocol for professional treatment: 2 min every day and 4 min once per week. After that, they were placed in a container with saline water and bicarbonate to simulate the oral environment. The pH was checked daily and maintained at 7.0.

Group treated with high-concentration fluoride product (G3): The teeth were totally immersed for 4 h in chlorinated water inside a container. After immersion, the teeth were rinsed and treated with a highly concentrated fluoride product. The product used was Elmex^®^ Dental Gel (Colgate-Palmolive Company, New York, NY, USA), containing 12.500 ppm F-. The gel was applied to one half of the enamel surface of each tooth for a period of 5 min. Then, they were carefully rinsed to remove the product. Afterwards, they were placed inside a container containing physiological water and bicarbonate. Again, the pH value of 7.0 was checked daily.

### 2.3. Statistical Analysis

*SPSS Statistics software 29.0.2.0 version* was utilized for data analysis. Descriptive analysis and frequency distribution were performed for the ETW indices. The results were analyzed using Wilcoxon’s test.

## 3. Results

The BEWE classifications of teeth surfaces included in the study at T1 (1 week), T2 (2 weeks), T3 (3 weeks), and T4 (4 weeks) are shown in Table 2, Table 3, Table 4 and Table 5.

**Figure 1:**
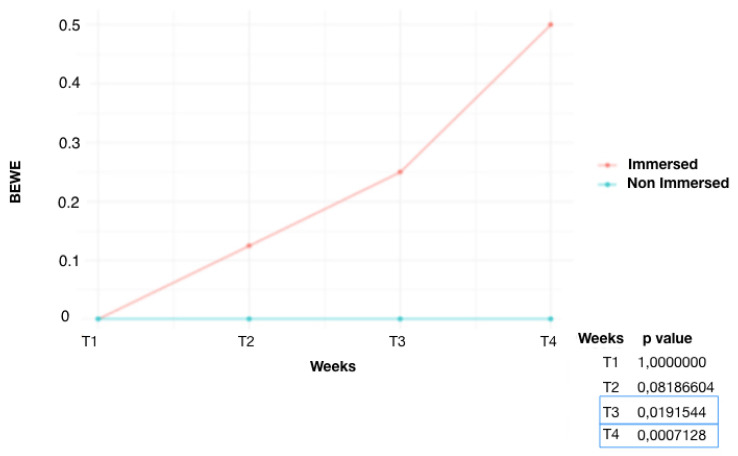
Average BEWE score over time, G1 (control group): comparison of immersed and non-immersed surfaces.

**Figure 2:**
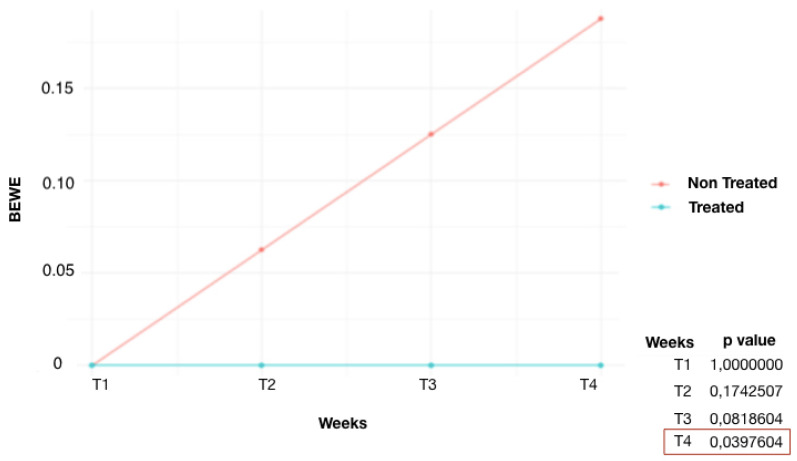
Average BEWE score over time, G2 (fluoride-treated group): comparison of treated and non-treated teeth.

**Figure 3:**
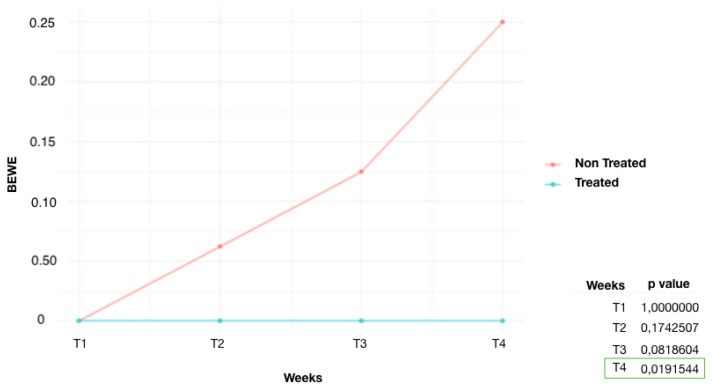
Average BEWE score over time, G3 (remineralization product-treated group): comparison of treated and non-treated teeth.

It is observed (Figure 1) that the control group (G1) started to show significant results of dental erosion on some enamel surfaces from week 3 (T_3_) (*p* = 0.019). The non-immersed part, on the other hand, showed no changes in enamel. At week 4 (T_4_), an increase in the number of eroded surfaces of the submerged teeth was detected (*p* = 0.0007). The fluoride-treated group (G2) (Figure 2) showed lower scores than G1. At week 4 (T_4_), the area treated with high-concentration fluoride products was not affected by tooth erosion, whereas the untreated area was only affected in three teeth. A significant difference was found at T_4_ (*p* = 0.039). Similar results were also found in the remineralization product-treated group (G3) (Figure 3). The tooth surfaces treated with remineralizing mousse showed no tooth erosion, whereas the untreated surfaces showed tooth erosion in only four teeth. Statistically significant evidence was found at week 4 (T_4_) (*p* = 0.019). 

## 4. Discussion

Swimming is the sport most frequently associated with the development of ETW because of the water pH decreasing due to the chlorine used to disinfect swimming pool water [11]. In the present in vitro study, the effects on the enamel surface of teeth immersed in chlorinated swimming pool water over a period of 1 month were investigated and the effectiveness of highly concentrated fluoride products and remineralizing products (F-APC) in preventing ETW was evaluated.

The effect of fluoride (i.e., the active agent of Elmex^®^Dental Gel) in the inhibition of the demineralization processes is well-known: it acts both on the reduction of metabolic and physiological pathways of acidogenic microorganisms and on the formation of fluorapatite, which is less soluble and more resistant to low pH values than pure hidroxyapatite (HA). Nevertheless, synthetic HA is an effective, widely described, and scientifically validated remineralizing agent too. It acts by inducing the physical restoration of the tooth surface, by releasing calcium and phosphate ions under acidic conditions and forming an interface between HA particles and the enamel [17]. The F-ACP complex (i.e., the active agent of Curasept Bioenamel Shock Action Mousse Caries Abrasion and Erosion) is a transient phase of natural hydroxyapatite formation, which releases a high concentration of calcium and phosphate ions in the oral cavity. In contact with saliva, the F-ACP complex rapidly and selectively releases the active substances (i.e., calcium, phosphate, and fluoride) in the areas of tooth hard tissues’ demineralization. Fluoride ions stabilize the ACP with the consequent rapid conversion of F-ACP into fluoroapatite [18].

In the three groups of teeth treated differently, different results were obtained. In the control group of untreated teeth immersed in chlorinated water, at T_1_ (one week), no elements showed erosion. This suggests that one week, with 4 h of immersion per day, is not enough time to develop ETW. The first changes were observed at T_2_ (two weeks), on two dental elements (BEWE index 1). At T_3_, ETW occurred on the surface of four elements. At T_4_, eight elements had erosion on the enamel surface. Thus, after one month, half of the submerged teeth were found to have ETW. It has been proven [19] that the swimmer’s exposure time to the erosive agent is directly proportional to the prevalence of roughened surfaces and tooth erosion. It was found that swimmers who trained for less than 2 h showed 20% ETW, while subjects who trained for more than 4 h showed 92.3% ETW. Regarding the years in which the sport has been practiced, there is evidence that the longer it is practiced, the more erosive lesions will be found [19].

In the fluoride-treated group, weekly, one half of every single tooth was treated with a product containing high-concentration fluoride, and the other half was not treated. In the treated tooth surfaces, no ETW was found at the enamel level during any evaluation. In the untreated surfaces, on the other hand, at the T_2_ evaluation (2 weeks), one element showed ETW. At the T_3_ evaluation, two elements, and at the T_4_ evaluation, three elements were affected by ETW. All erosions were assigned the BEWE index value 1. The preventive effects of fluoride have been linked to the formation of precipitates on the tooth surface, which act as a protective barrier against acid impact [20]. At the T_4_ assessment, the number of elements affected by ETW was significantly lower than in the chlorine group. That is because the fluoride product applied to a part of the tooth surface is able to protect both the treated and untreated parts of the tooth surface. This is due to the property of amine fluoride, which is able to distribute evenly over the enamel surface, forming a stable, calcium fluoride-rich layer that prolongs fluoride and mineral deposition over time [21,22]. 

In the remineralization product-treated group, half of each tooth was treated daily with a remineralizing product. From the initial evaluation T_0_ to the T_4_ evaluation, it emerged that the treated tooth surfaces did not develop ETW. On the other hand, in the untreated tooth surfaces, one element showed ETW at T_2_, two elements at T_3_, and four elements at T_4_. It is possible to observe that the number of untreated eroded teeth in this group was lower than the eroded teeth in the control group. These can be explained by the daily application of the product, which was transferred to a salivary environment, making it rich in minerals [23]. It was also deposited on the untreated surfaces and subsequently remineralized them [24].

Chlorinated pool water is thus a potential erosive agent for teeth if the water pH values reach a critical value. It is therefore essential to prevent ETW through dental examinations and appropriate preventive treatments. It has been found that many professional swimmers claim that they do not adhere to constant monitoring and do not receive dental treatment [25]. To remedy this deficiency of information and awareness among athletes, more attention and time should be invested in educating athletes and athletic trainers. Also, the dental team’s role is important: to intercept the problem early, recommend preventive treatments, and schedule regular recalls [26].

A future research idea is to repeat the study in vivo, by testing the different remineralizing products both for preventive and therapeutic purposes among swimmers, even with a split mouth design. Furthermore, as different swimming pools can present different levels of water chlorination, it would be useful to conduct a multicenter study, i.e., by enrolling the sample of swimmers in various swimming pools of different geographic areas. The future prospect is to define a protocol for the prevention and treatment of dental erosion in competitive swimmers, which clinician of both the public and the private sector can recommend to their “swimmer patients” and use to treat them.

## 5. Conclusions

Chlorinated pool water is a potential erosive agent for teeth if water pH values reach a critical value. The time spent by the swimmer in the water exercising is a significant factor in tooth damage. Treating the teeth surface with highly concentrated fluoride products, once a week for 5 min, is effective in protecting against tooth erosion. Remineralizing products such as F-APC, applied once a day for 2 min and once a week for 4 min, are effective in protecting tooth surfaces from ETW.

## Figures and Tables

**Table 1:** Basic Erosive Wear Examination (BEWE) [16].

Score	Description
0	No erosive tooth wear (no surface loss)
1	Initial loss of enamel surface texture
2 ^a^	Distinct defect, hard tissue loss (dentine) <50% of the surface area
3 ^a^	Hard tissue loss >50% of the surface area

^a^ in scores 2 and 3, dentine often is involved.

**Table 2:** BEWE classifications of teeth surfaces included in the study at T1 (1 week) divided by treatment groups.

*G1*		*Immersed Surfaces*	*G2*		*Treated Surfaces*	*G3*		*Treated Surfaces*
1	0	0	1	0	0	1	0	0
2	0	0	2	0	0	2	0	0
3	0	0	3	0	0	3	0	0
4	0	0	4	0	0	4	0	0
5	0	0	5	0	0	5	0	0
6	0	0	6	0	0	6	0	0
7	0	0	7	0	0	7	0	0
8	0	0	8	0	0	8	0	0
9	0	0	9	0	0	9	0	0
10	0	0	10	0	0	10	0	0
11	0	0	11	0	0	11	0	0
12	0	0	12	0	0	12	0	0
13	0	0	13	0	0	13	0	0
14	0	0	14	0	0	14	0	0
15	0	0	15	0	0	15	0	0
16	0	0	16	0	0	16	0	0

**Table 3:** BEWE classifications of teeth surfaces included in the study at T2 (2 weeks) divided by treatment groups.

*G1*		*Immersed Surfaces*	*G2*		*Treated Surfaces*	*G3*		*Treated Surfaces*
1	0	1	1	0	0	1	0	0
2	0	0	2	0	0	2	0	0
3	0	0	3	0	0	3	0	0
4	0	0	4	0	0	4	0	0
5	0	0	5	1	0	5	0	0
6	0	0	6	0	0	6	0	0
7	0	0	7	0	0	7	1	0
8	0	0	8	0	0	8	0	0
9	0	0	9	0	0	9	0	0
10	0	0	10	0	0	10	0	0
11	0	0	11	0	0	11	0	0
12	0	0	12	0	0	12	0	0
13	0	0	13	0	0	13	0	0
14	0	0	14	0	0	14	0	0
15	0	1	15	0	0	15	0	0
16	0	0	16	0	0	16	0	0

**Table 4:** BEWE classifications of teeth surfaces included in the study at T3 (3 weeks) divided by treatment groups.

*G1*		*Immersed Surfaces*	*G2*		*Treated Surfaces*	*G3*		*Treated Surfaces*
1	0	1	1	0	0	1	0	0
2	0	1	2	0	0	2	0	0
3	0	0	3	0	0	3	1	0
4	0	0	4	0	0	4	0	0
5	0	0	5	1	0	5	0	0
6	0	0	6	0	0	6	0	0
7	0	0	7	0	0	7	1	0
8	0	1	8	0	0	8	0	0
9	0	0	9	0	0	9	0	0
10	0	0	10	0	0	10	0	0
11	0	1	11	0	0	11	0	0
12	0	0	12	0	0	12	0	0
13	0	0	13	1	0	13	0	0
14	0	0	14	0	0	14	0	0
15	0	0	15	0	0	15	0	0
16	0	0	16	0	0	16	0	0

**Table 5:** BEWE classifications of teeth surfaces included in the study at T4 (4 weeks) divided by treatment groups.

*G1*		*Immersed Surfaces*	*G2*		*Treated Surfaces*	*G3*		*Treated Surfaces*
1	0	1	1	0	0	1	0	0
2	0	1	2	0	0	2	0	0
3	0	0	3	0	0	3	1	0
4	0	0	4	0	0	4	1	0
5	0	0	5	1	0	5	0	0
6	0	0	6	1	0	6	1	0
7	0	1	7	0	0	7	1	0
8	0	1	8	0	0	8	0	0
9	0	0	9	0	0	9	0	0
10	0	1	10	0	0	10	0	0
11	0	1	11	0	0	11	0	0
12	0	0	12	0	0	12	0	0
13	0	1	13	1	0	13	0	0
14	0	1	14	0	0	14	0	0
15	0	0	15	0	0	15	0	0
16	0	0	16	0	0	16	0	0

## Data Availability

The raw data supporting the conclusions of this article will be made available by the authors on request.

## References

[1] Buczkowska-Radlińska J., Łagocka R., Kaczmarek W., Górski M., Nowicka A. (2013). Prevalence of dental erosion in adolescent competitive swimmers exposed to gas-chlorinated swimming pool water. Clin. Oral Investig..

[2] Abdelrahman H.H., Ammar N., Hassan M.G., Essam W., Amer H. (2023). Erosive tooth wear and salivary parameters among competitive swimmers and non-swimmers in Egypt: A cross-sectional study. Clin. Oral Investig..

[3] Carvalho T.S., Colon P., Ganss C., Huysmans M.C., Lussi A., Schlueter N., Schmalz G., Shellis R.P., Tveit A.B., Wiegand A. (2015). Consensus report of the European Federation of Conservative Dentistry: Erosive tooth wear—Diagnosis and management. Clin. Oral Investig..

[4] Johansson A.K., Omar R., Gunnar E., Carlsson G.E., Johansson A. (2012). Dental Erosion and Its Growing Importance in Clinical Practice: From Past to Present. Int. J. Dent..

[5] Lussi A., Schlueter N., Rakhmatullina E., Ganss C. (2011). Dental Erosion—An Overview with Emphasis on Chemical and Histopathological Aspects. Caries Res..

[6] Lussi A., Jaeggi T. (2008). Erosion—Diagnosis and risk factors. Clin. Oral Investig..

[7] Yanushevich O.O., Maev I.V., Krikheli N.I., Andreev D.N., Lyamina S.V., Sokolov F.S., Bychkova M.N., Beliy P.A., Zaslavskaya K.Y. (2022). Prevalence and Risk of Dental Erosion in Patients with Gastroesophageal Reflux Disease: A Meta-Analysis. Dent. J..

[8] Otsu M., Hamura A., Ishikawa Y., Karibe H., Ichijyo T., Yoshinaga Y. (2014). Factors affecting the dental erosion severity of patients with eating disorders. Biopsychosoc. Med..

[9] Hemalatha V.T., Manigandan T., Sarumathi T., Aarthi Nisha V., Amudhan A. (2013). Dental Considerations in Pregnancy—A Critical Review on the Oral Care. J. Clin. Diagn. Res..

[10] Nijakowski K., Zdrojewski J., Nowak M., Podgórski F., Surdacka A. (2022). Regular Physical Activity and Dental Erosion: A Systematic Review. Appl. Sci..

[11] Puleio F., Di Spirito F., Lo Giudice G., Pantaleo G., Rizzo D., Lo Giudice R. (2023). Long-Term Chromatic Durability of White Spot Lesions through Employment of Infiltration Resin Treatment. Medicina.

[12] Zebrauskas A., Birskute R., Maciulskiene V. (2014). Prevalence of Dental Erosion among the Young Regular Swimmers in Kaunas, Lithuania. J. Oral Maxillofac. Res..

[13] Salem M.N., Hafez S. (2021). Aesthetic Management of Erosive Tooth Wear in a Young Egyptian Swimmer: A Case Report. Clin. Cosmet. Investig. Dent..

[14] Sirimaharaj V., Brearley Messer L., Morgan M.V. (2002). Acidic diet and dental erosion among athletes. Aust. Dent. J..

[15] Bartlett D., Ganss C., Lussi A. (2008). Basic Erosive Wear Examination (BEWE): A new scoring system for scientific and clinical needs. Clin. Oral Investig..

[16] A Plea for the Development of an Universally Accepted Modular Tooth Wear Evaluation System—Scientific Figure on ResearchGate. https://www.researchgate.net/figure/Basic-Erosive-Wear-Examination-BEWE-8_tbl3_309693771.

[17] Degli Esposti L., Ionescu A.C., Brambilla E., Tampieri A., Iafisco M. (2020). Characterization of a Toothpaste Containing Bioactive Hydroxyapatites and In Vitro Evaluation of Its Efficacy to Remineralize Enamel and to Occlude Dentinal Tubules. Materials.

[18] Sathish A.K., Gopalkrishna P., Kumar S. (2023). In vitro evaluation of remineralizing agents on dentinal tubule occlusion: A scanning electron microscopic study. J. Ind. Soc. Periodontol..

[19] Baghele O.N., Majumdar I.A., Thorat M.S., Nawar R., Baghele M.O., Makkad S. (2013). Prevalence of dental erosion among young competitive swimmers: A pilot study. Compend. Contin. Educ. Dent..

[20] Monterubbianesi R., Sparabombe S., Tosco V., Profili F., Mascitti M., Hosein A., Putignano A., Orsini G. (2020). Can Desensitizing Toothpastes Also Have an Effect on Gingival Inflammation? A Double-Blind, Three-Treatment Crossover Clinical Trial. Int. J. Environ. Res. Public Health.

[21] Wiegand A., Bichsel D., Magalhães A.C., Becker K., Attin T. (2009). Effect of sodium, amine and stannous fluoride at the same concentration and different pH on in vitro erosion. J. Dent..

[22] Mazzoleni S., Gargani A., Parcianello R.G., Pezzato L., Bertolini R., Zuccon A., Stellini E., Ludovichetti F.S. (2023). Protection against Dental Erosion and the Remineralization Capacity of Non-Fluoride Toothpaste, Fluoride Toothpaste and Fluoride Varnish. Appl. Sci..

[23] Tosco V., Vitiello F., Monterubbianesi R., Gatto M.L., Orilisi G., Mengucci P., Putignano A., Orsini G. (2023). Assessment of the Remineralizing Potential of Biomimetic Materials on Early Artificial Caries Lesions after 28 Days: An In Vitro Study. Bioengineering.

[24] Ludovichetti F.S., Zambon G., Cimolai M., Gallo M., Signoriello A.G., Pezzato L., Bertolini R., Mazzoleni S. (2022). Efficacy of Two Toothpaste in Preventing Tooth Erosive Lesions Associated with Gastroesophageal Reflux Disease. Appl. Sci..

[25] Cardoso F., Monteiro A.S., Fernandes A., Vilas-boas J.P., Pinho J.C., Pyne D., Ricardo J., Fernandes R.J. (2020). Oral health in young elite swimmers. Trends Sport Sci..

[26] Engineer S., Tamgadge S., Rijhwani N., Choudhary N., Shukla G. (2022). Awareness of dental health in competitive and recreational swimmers: A comparative survey-based study. J. Datta Meghe Inst. Med. Sci. Univ..

